# Acute Effects of Using Added Respiratory Dead Space Volume in a Cycling Sprint Interval Exercise Protocol: A Cross-Over Study

**DOI:** 10.3390/ijerph17249485

**Published:** 2020-12-18

**Authors:** Natalia Danek, Kamil Michalik, Marcin Smolarek, Marek Zatoń

**Affiliations:** 1 Department of Physiology and Biochemistry, Faculty of Physical Education and Sport, University School of Physical Education in Wroclaw, 51-612 Wroclaw, Poland; marcin.cluby@interia.eu (M.S.); marek.zaton@awf.wroc.pl (M.Z.); 2 Department of Human Motor Skills, Faculty of Physical Education and Sport, University School of Physical Education in Wroclaw, 51-612 Wroclaw, Poland; kamil.michalik@awf.wroc.pl

**Keywords:** sprint interval exercise, cardiorespiratory responses, blood lactate, added respiratory dead space, respiratory acidosis

## Abstract

*Background:* The aim of the study was to compare acute physiological, biochemical, and perceptual responses during sprint interval exercise (SIE) with breathing through a device increasing added respiratory dead space volume (ARDS_V_) and without the device. *Methods:* The study involved 11 healthy, physically active men (mean maximal oxygen uptake: 52.6 ± 8.2 mL∙kg^1^∙min^−1^). During four visits to a laboratory with a minimum interval of 72 h, they participated in (1) an incremental test on a cycle ergometer; (2) a familiarization session; (3) and (4) cross-over SIE sessions. SIE consisted of 6 × 10-s all-out bouts with 4-min active recovery. During one of the sessions the participants breathed through a 1200-mL ARDSv (SIE_ARDS_). *Results:* The work performed was significantly higher by 4.4% during SIE_ARDS_, with no differences in the fatigue index. The mean respiratory ventilation was significantly higher by 13.2%, and the mean oxygen uptake was higher by 31.3% during SIE_ARDS_. Respiratory muscle strength did not change after the two SIE sessions. In SIE_ARDS_, the mean pH turned out significantly lower (7.26 vs. 7.29), and the mean HCO_3_^–^ concentration was higher by 7.6%. Average La^−^ and rating of perceived exertion (RPE) did not differ between the sessions. *Conclusions:* Using ARDS_V_ during SIE provokes respiratory acidosis, causes stronger acute physiological responses, and does not increase RPE.

## 1. Introduction

Interval training can be described as intermittent high-intensity exercises divided by periods of incomplete recovery (e.g., lower-intensity work) [1]. Among its most popular types, there is sprint interval training (SIT), which consists in performing maximum-intensity work (generating the highest possible power, the so-called “all-out” training) [2]. A single session normally consists of two to six efforts of 10–30-s, with recovery lasting longer (e.g., several minutes) and a usual total session time of 10–30-min [3]. With reference to the generally recommended moderate-intensity continuous training, SIT is considered an effective and time-efficient strategy for improving general physical capacity and cardiorespiratory capacity (e.g., maximal oxygen uptake (VO_2_max)), as well as lowering the risk of cardiometabolic diseases in the healthy population [4]. A study by Hazell et al. [5] based on a 2-week training program revealed that 10-s efforts (with 4-min recovery) efficiently improved cardiorespiratory capacity as compared with the “classic” SIT protocol, involving 4–6 × 30-s efforts with 4-min rest. However, solutions are still being sought that will increase the training effects without additional time expenditure.

Several studies have analyzed, during single effort or regular training, the effects of applying modifications of inhaled air composition by using various types of gas mixtures, e.g., increasing the amount of carbon dioxide (CO_2_) in the inhaled air [6], training masks increasing respiratory resistance [7,8], or added respiratory dead space volume (ARDS_V_) [9,10]. Compared to the Elevation Training Mask^®^ (ETM) (Training Mask LLC, Cadillac, Michigan) or an airflow restriction mask (ARM) [7,8], a device used to increase respiratory dead space volume has an additional corrugated pipe of a certain length, but it has no valves to increase breathing resistance [9,10]. With reference to this latest area, research is available in which ARDS_V_ was used during continuous effort of constant intensity [11] and during progressive effort [12]. Several experiments have confirmed the effectiveness of 1000-mL ARDS_V_ in developing physical capacity (as reflected in VO_2_max) in regular high-intensity interval training among swimmers [13] and triathletes [14]. On the other hand, 30-min training at a level of 60% VO_2_max performed for 6 weeks (twice a week) with ARDS_V_ of 1000–1200-mL did not improve VO_2_max in young physically active males [15] or in swimmers [9]. This suggests that there may be a specific effort intensity that conditions achieving the desired adaptive changes during ARDS_V_ application. Thus, it is necessary to examine acute physiological, biochemical, and psychological responses during a single sprint interval exercise (SIE) session and to compare the results with those obtained under standard conditions to determine whether this approach can provide a stronger training stimulus. This will help plan regular training, especially for individuals who make intensive efforts in conditions of increased CO_2_ concentration in the inhaled air, e.g., divers, firefighters, miners, or astronauts.

Breathing with ARDS_V_ leads to CO_2_ accumulation and an increase in its partial pressure in arterial (PaCO_2_) and venous blood (pCO_2_) [11,16]. It was found that PaCO_2_ could be determined in an ergospirometry test by using end-tidal partial pressure of CO_2_ (P_ET_CO_2_) [17]. Increased partial pressure of CO_2_ in blood (>45 mm Hg) results in high concentrations of hydrogen ions (H^+^), low pH (<7.35), and raised bicarbonate (HCO_3_^–^) concentration (>30 mm Hg), which is a state referred to as respiratory acidosis or hypercapnic acidosis [18]. In response to increased blood CO_2_ levels, chemoreceptors provide respiratory feedback [19] by raising respiratory ventilation (VE) through its components: tidal volume (VT) and respiratory frequency (Rf) [20]. Higher VE is required to maintain PaCO_2_ and H^+^ regulation with any metabolic rate [16]. It has been proved that higher VE leads to increased oxygen uptake (VO_2_) as a result of higher respiratory muscle activity [6,8]. In turn, an increased effort of the respiratory muscles can lead to their fatigue. It has been suggested that changing the ventilation pattern can support respiratory muscle training (RMT). Moreover, increased VE, especially through a rise in Rf, can increase the rating of perceived exertion (RPE) [21]. From a psychological point of view, understanding these responses is important, as protocols that are better perceived (lower RPE) are more frequently chosen during regular training. Additionally, the lowering of blood pH influences the rate of glycogenolysis, glycolysis, and lactic acid production, as well as the functioning of monocarboxylate transporter 1 (MCT1) across sarcolemma [22]. It is suggested that hypercapnia can reduce lactate (La^−^) release from muscles to blood [23,24].

So far, no study has compared actual responses during a single SIE session with ARDS_V_ breathing. Therefore, the aim of this research was to determine the physiological and biochemical responses and RPE during a single SIE session consisting of 6 × 10-s bouts with an active 4-min rest interval with 1200-mL ARDS_V_ breathing, as well as to compare the responses with those obtained during a session performed under standard conditions without breathing impediments. We assumed that the application of ARDS_V_ and inhaling increased CO_2_ concentrations would cause hypercapnic acidosis, which, in turn, would initiate deeper changes in the acid–base balance and reduction of blood La^−^ concentration. According to another hypothesis, this would increase the respiratory system activity, as reflected in higher VO_2_. In addition, we tested a hypothesis that higher exercise VE would provoke greater respiratory muscle fatigue.

## 2. Materials and Methods

### 2.1. Participants

The study involved 11 healthy, physically active males who volunteered to participate. Each of them declared a minimum of 5 h per week of physical exercise (sports classes at a university, gym, volleyball, football, running). No participant practiced sport at a professional level or was classified in a risk group for respiratory, cardiovascular, or metabolic diseases. They did not have experience with regular cycling training. There were no smokers among the participants. All became familiar with the study procedure and provided a written informed consent to participate. The study was approved by the University Research Ethics Committee (1/2019) and followed the tenets of the Declaration of Helsinki (PN-EN ISO 9001:2001 certificate). Detailed characteristics of the respondents are presented in Table 1.

### 2.2. Study Design

The research included 4 visits to a laboratory with an interval of at least 72 h. During the visits, sessions of exercises on a cycle ergometer were conducted. All sessions were supervised by the same investigators and performed in the morning, 2 h after breakfast. During the experiment, the participants maintained physical activity patterns and were to refrain from exercise, alcohol, and caffeine for 24 h before each laboratory session. During the first visit, body mass (kg) and body height (cm) were measured with WPT 200 medical scales (Radwag, Radom, Poland); resting arterial blood pressure was evaluated with an aneroid sphygmomanometer (Riester, Jungingen, Germany); also, a spirometry test and an incremental exercise test (IET) were performed to determine cardiorespiratory capacity. The second visit included familiarization with the cycling SIE protocol and with ARDS_V_ breathing. During the third and fourth visits, cross-over SIE sessions were conducted in a random order: the participants were breathing under standard conditions in one session and with ARDS_V_ in another session, and subsequently the other way round.

### 2.3. Spirometry Test

The spirometry test was performed by using a Quark b^2^ ergospirometer (Cosmed, Milan, Italy). It involved an inspiration with a maximum volume preceded by 2, 3 quiet breaths and ended with an intense exhalation with a maximum airflow, resulting in a minimum volume of residual air. In the course of the respiratory test, the following parameters were recorded: peak expiratory flow (PEF), peak inspiratory flow (PIF), forced vital capacity (FVC), and forced expiratory volume in 1 s (FEV_1_). Each participant took 3 trials, with the first one for familiarization. Tiffeneau index (FEV_1_ · FVC^−1^) was calculated by the dedicated software.

### 2.4. Incremental Exercise Test (IXT)

This was performed on an Excalibur Sport cycle ergometer (Lode BV, Groningen, Netherlands) in accordance with a ramp incremental test protocol with a linear load pattern. The test started with a load of 0 W, which increased by ca. 0.28 W every second [25]. The pedaling frequency of above 60 rpm was maintained. The tested person was breathing through a mask, and the expired air was analyzed by a Quark b^2^ device (Cosmed, Milan, Italy). Before the examination, the device was calibrated with atmospheric air and a gas mixture composed of 5% CO_2_, 16% O_2_, and 79% N_2_. Breathing parameters were recorded breath by breath. VE, Rf, VT, and VO_2_ were measured, and the results were averaged every 30-s and converted to minute values. Heart rate (HR) was determined with a S810 sport tester (Polar Electro, Kempele, Finland) and recorded by the Quark b^2^ analyzer software. VO_2_max was registered as the highest 30-s average value at a plateau of VO_2_ < 1.35 mL∙kg^1^∙min^−1^ despite the increasing load or if at least 2 of the following criteria were met: (1) volitional exhaustion, (2) predicted HRmax ≥95% (220—age), (3) respiratory rate ≥1.15. Maximal workload (Wmax) was determined as power at the end of the test. Capillary blood was collected from a hand fingertip to heparinized capillaries in the third minute after the test to determine La^−^ concentration in a photometer (LP 400, Dr. Lange, Berlin, Germany). RPE was evaluated with the use of the Borg scale [26] immediately after the test.

### 2.5. Cycling Sprint Interval Exercise Sessions

**Figure 1:**
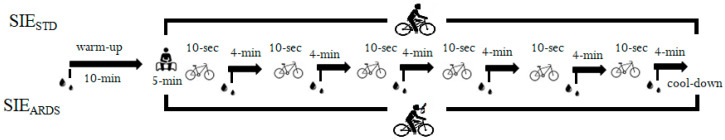
Cycling sprint interval exercise (SIE) protocol SIE_STD_—standard protocol, SIE_ARDS_—protocol with the added respiratory dead space volume.

**Figure 2:**
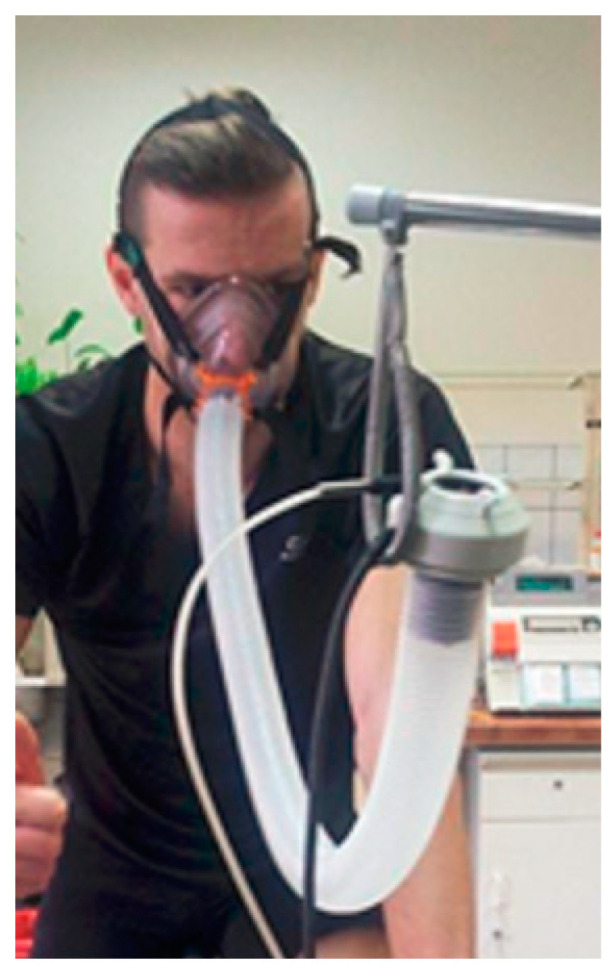
One of the participants with the added respiratory dead space volume (ARDS_V_) device.

Both SIE sessions were performed on a cycle ergometer (Ergomedic Monark 894, Vansbro, Sweden) in accordance with the protocol described by Danek et al. [27] and presented in Figure 1. Each session was preceded by a 10-min warm-up at 60% VO_2_max obtained in the incremental test; during the warm-up, two 5-s all-out accelerations were performed in the third and sixth minute. The warm-up was followed by a 5-min rest in a sitting position. In the main study part, the participants conducted 6 × 10-s bouts with an individual load of 7.5% of the body mass and an active break of 4-min with a load of 50 W and frequency of 50 rpm. Standardized verbal encouragement for each participant was provided throughout the during the bouts. During one SIE session, ARDS_V_ (SIE_ARDS_) (Figure 2) was applied after warm-up, two min before the first bout, and removed after the cool-down. The total time of ARDS_V_ application equaled 27-min. The second session was performed under standard conditions without ARDS_V_ (SIE_STD_).

The Quark b^2^ analyzer (Cosmed, Milan, Italy) measured VE and its components: Rf and VT, VO_2_, expired CO_2_ (VCO_2_), fraction of inspired oxygen (FiO_2_), fraction of inspired CO_2_ (FiCO_2_), end-tidal partial pressure of oxygen (P_ET_O_2_), P_ET_CO_2_, time of inspiration (Ti), time of expiration (Te), total time of the respiratory cycle (Ttot), and the ratio of inspiration time and respiratory cycle time (Ti · Ttot^−1^). The software also calculated ventilatory equivalents for oxygen (VE · VO_2_^−1^) and for CO_2_ (VE · VCO_2_^−1^), as well as oxygen pulse (VO_2_ · HR^−1^). HR was determined with a S810 sport tester (Polar Electro, Kempele, Finland). The results were averaged every 30-s and converted to minute values.

Before and after SIE, inspiratory muscle strength (maximal inspiratory pressure, PImax) and expiratory muscle strength (maximal expiratory pressure, PEmax) were measured with a Micro RPM device (CareFusion, San Diego, CA, USA). To assess PImax, the tested person, in a standing position, performed a maximum inspiration from the level of a maximum expiration. Then, to evaluate PEmax, the individual exhaled starting from the maximum inspiration level. In both cases, a special nose stopper was fitted. Each participant took 3 trials each time, and the highest recorded values were selected for further analysis.

Capillary blood was collected from a hand fingertip to heparinized capillaries in the third minute after each bout to determine blood acid–base balance: pH, pCO_2_, current HCO_3_^–^ concentration, and blood oxygen saturation (SaO_2_) with the use of a RapidLab 348 analyzer (Bayer, Germany), as well as La^−^ concentration in a photometer (LP 400, Dr. Lange, Berlin, Germany).

### 2.6. Performance

The results obtained during both SIE sessions were analyzed with the consideration of peak power output (PPO), mean power output (MPO), and total work (Wtot). These parameters were calculated with the MCE 2.0 software (MCE, Wroclaw, Poland) for 6 repetitions. Fatigue level was estimated by calculating the fatigue index (FI) with the following formula: [100 × (total sprint MPO × ideal sprint MPO^−1^)]—100; where total sprint MPO—sum of sprint MPO from all sprints, ideal sprint MPO—number of sprints (6) × the highest sprint MPO. This formula has been recognized as the most valid and reliable method for assessing fatigue in multiple sprint tests [28].

### 2.7. Device Added Respiratory Dead Space Volume (ARDS_V_)

ARDS_V_ was created by a single-valve ambu-type mask and an attached 2.5-cm diameter ribbed snorkel to provide of 1200-mL total volume. Dead space volume was identical for each participant and measured by filling the snorkel with water and then transferring the volume to a graduated cylinder, as described by Szczepan et al. [9]. 

### 2.8. Statistical Analysis

The sample size was established a priori by using G*Power 3.1 software (v3.1.9.2, Kiel, Germany) [29]. The expected effect size (ES) was set at (Cohen’s f) 0.85, the α level was set at 0.05, and the power (1-β) was set at 0.8 [30]. The 11 participants in the group were necessary and finally recruited.

The average values of cardiopulmonary parameters in both SIE sessions were calculated for 25-min (1-min of work, 20-min recovery, and 4-min of cool-down). The mean values of pH, La^−^, current HCO_3_^–^ concentration, and RPE were determined on the basis of measurements taken after each of the 6 bouts.

The statistical analysis of the data was performed with the Statistica 13.3 software (StatSoft Inc., Tulsa, OK, USA). All of the results are presented as arithmetic means ± standard deviations ($\bar{x}$ ± SD). The Shapiro–Wilk test was applied to assess the normality of the tested characteristics distribution, and the Levene’s test evaluated the equality of variances. The Student’s t-test for dependent samples served to evaluate the differences of selected variables between the SIE protocols. A two-way (protocol × number of bouts) analysis of variance (ANOVA) with repeated measures was used to compare PPO, pCO_2_, and RPE. When a significant F ratio value was obtained, the Bonferroni post-hoc test was performed. The level of *p* < 0.05 was assumed statistically significant. ES, or Cohen’s d, was calculated in order to show the practical effect, with the following criteria: 0.1—trivial, 0.2—small, 0.5—medium, 0.8—large [30].

## 3. Results

In the incremental exercise test, the studied individuals achieved the following results: Wmax: 336.9 ± 40.9 W, VEmax: 148.8 ± 22.1 L · min^−1^, Rfmax: 51.0 ± 7.9 L · min^−1^, VTmax: 3.4 ± 0.5 L, VO_2_max: 52.6 ± 8.2 mL · kg^−1^ · min^−1^, HRmax: 193 ± 7 beats · min^−1^, La^−^peak_IET_: 12.9 ± 1.8 mmol · L^−1^, RPE_IET_: 19.0 ± 0.9.

**Figure 3:**
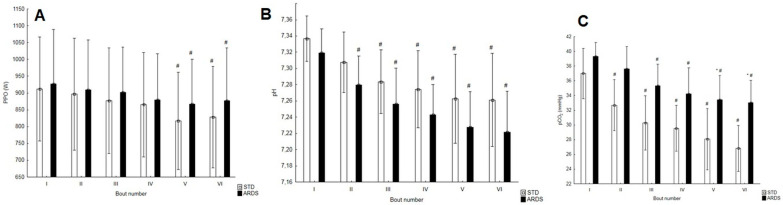
Changes in (**A**) peak power output (PPO), (**B**) pH values, and (**C**) carbon dioxide partial pressure (pCO_2_) in the subsequent bouts during SIE sessions. Statistically significant difference as compared with the first bout (*p* < 0.05), # Statistically significant difference as compared with the first bout (*p* < 0.05), * Statistically significant difference between the SIE protocols.

PPO did not differ statistically significantly between the bouts in the SIE protocols (Figure 3A). In both protocols, the values in the fifth and sixth bout were statistically significantly lower than those in the first repetition (Figure 3A). The mean power turned out statistically significantly higher in SIE_ARDS_ (787.6 ± 139.1 W) as compared with SIE_STD_ (754.8 ± 132.7 W) (*p* < 0.01, t = 3.98, ES = 0.24). The amount of work performed differed statistically significantly between the protocols (*p* < 0.01, t = 3.98, ES = 0.24) and equaled 45.3 ± 8.0 kJ and 47.3 ± 8.3 kJ, respectively, in SIE_STD_ and SIE_ARDS_. No statistically significant difference was observed with regard to FI (*p* = 0.10, t = 1.82).

Peak La^−^ concentration amounted to 13.9 ± 1.9 mmol · L^−1^ in SIE_STD_ and 12.8 ± 1.5 mmol · L^−1^ in SIE_ARDS_ and did not significantly differ between the sessions (*p* = 0.09, t = 1.89). Mean La^−^ concentration after 6 bouts did not differ between the protocols, either (*p* = 0.08, t = 1.95); it equaled 11.0 ± 1.2 mmol · L^−1^ and 10.2 ± 1.2 mmol · L^−1^, respectively, in SIE_STD_ and SIE_ARDS_. Both of them (peak and mean La^−^ values) were close to assuming the threshold equal 0.05 to reject the null hypothesis. The value of pH did not differ statistically significantly in subsequent bouts between the protocols. In SIE_ARDS_, starting with the second bout, it was statistically significantly lower in each subsequent bout than in the first one (*p* < 0.001); in SIE_STD_, the same was observed starting with the third bout (*p* < 0.001) (Figure 3B). Mean pH was statistically significantly lower (*p* < 0.01, t = 3.54, ES = 0.77) in SIE_ARDS_ (7.26 ± 0.04) than in SIE_STD_ (7.29 ± 0.04). The blood partial pressure of CO_2_ differed statistically significantly after the fifth (*p* < 0.05) and sixth bout (*p* < 0.01). The value of pCO_2_ lowered in the subsequent bouts in both protocols, beginning with the second one in SIE_STD_ and with the third one in SIE_ARDS_ (Figure 3C). Mean current HCO_3_^–^ concentration differed between the protocols and was statistically significantly higher (*p* < 0.05, t = 3.09, ES = 0.74) during SIE_ARDS_ (15.6 ± 1.6 mmol · L^−1^) as compared with SIE_STD_ (14.5 ± 1.5 mmol · L^−1^). SaO_2_ did not differ statistically significantly between the investigated conditions, remaining within the physiological norm of 95–98%.

The highest RPE equaled 17.6 ± 1.4 in SIE_STD_ and 18.0 ± 1.4 in SIE_ARDS_ and did not differ statistically significantly (*p* = 0.42, t = 0.84). Mean RPE for the 6 bouts did not differ between the protocols (*p* = 0.40, t = 0.89) and totaled 15.2 ± 1.0 in SIE_STD_ and 14.9 ± 0.7 in SIE_ARDS_.

**Figure 4:**
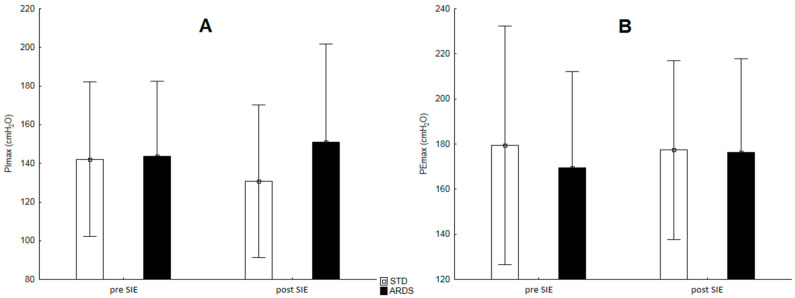
Changes in maximal inspiratory (**A**) and expiratory (**B**) muscle strength before and after the SIE protocols.

The inspiratory or expiratory muscle strength did not change in either SIE protocol. In addition, it did not differ statistically significantly between the applied conditions (Figure 4).

**Figure 5:**
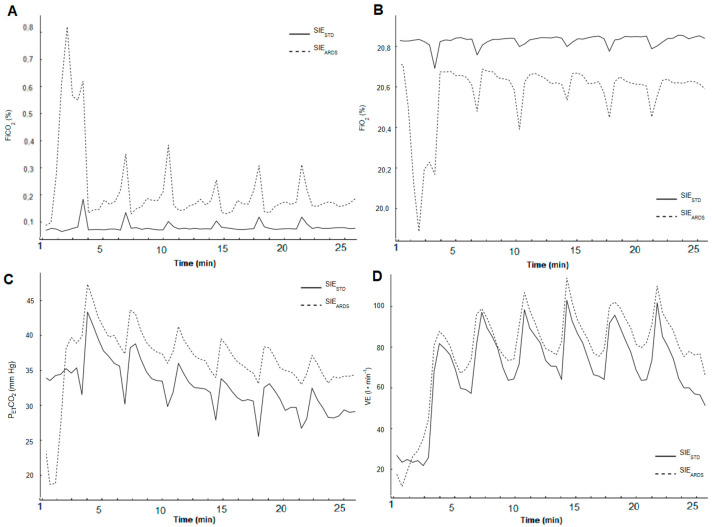
The average values of (**A**) FiO_2_, (**B**) FiCO_2_, (**C**) P_ET_CO_2_, and (**D**) ventilation (VE)—two minutes before, during and four minutes after last bout in SIE_STD_ and SIE_ARDS_ (27 min total).

The mean physiological parameters in both SIE protocols are compared in Table 2. The average values of VE, FiO_2_, FiCO_2_, and P_ET_CO_2_ kinetics during both SIE protocols are shown in Figure 5.

## 4. Discussion

We examined the effect of ARDS_V_ breathing during a single SIE session and compared it with the results obtained during a session without ARDS_V_. This is the first study to verify this approach. The achieved results confirmed our first hypothesis because during SIE_ARDS_, the respiratory system activity was increased. However, this did not cause any major respiratory muscle fatigue. The application of ARDS_V_ lowered blood pH as compared with SIE_STD_, but this did not contribute to a statistically significant decrease in La^−^ concentration or increase in RPE.

When considering the effect of 1200-mL ARDS_V_ breathing, attention should be paid to the induced changes in the inhaled air composition, as this has not been reported in the previous studies. It is known that ARDS_V_ breathing raises FiCO_2_ [31]; however, there are no results of studies on the amplitude of changes in the inhaled air composition during an exercise session, as applied in the experiment that we presented. We found that during SIE_ARDS_, there was a slight decrease in FiO_2_ and a slight increase in FiCO_2_. Nevertheless, we did not observe hypoxia or a significant reduction in the oxygen content in the inhaled air or blood. Similar conclusions were presented by Barbieri et al. [8]: their research participants breathed an airflow restriction mask by means of special valves, which increased the respiratory dead space by 350-mL. Therefore, neither device can simulate high-altitude training. The level of inhaled CO_2_ in own research was lower compared with other research, in which gas mixtures containing >2% CO_2_ in the inhaled air were used [6,32]. Thus, it would seem that inhaling air containing an average of 0.19% CO_2_ would not lead to significant changes in work efficiency, acid–base balance, or current physiological responses of the body.

No reduction in effort efficiency occurred during SIE_ARDS_. Maintaining a high level of maximum power in subsequent bouts is important for long-term training adaptations, as reported by Hazell et al. [5]. Although the maximum power did not differ between conditions in any bout, the sum of the work performed was statistically significantly higher when breathing with ARDS_V_. To the best of our knowledge, there are no research results that would diagnose the effect of inhaling CO_2_ on work efficiency during maximum sprinting efforts on a cycle ergometer; therefore, it is difficult to relate our results to other authors’ studies. Therefore, possible explanations for this phenomenon should be considered on the basis of the obtained research results. It is probable that the increased oxygen availability (higher average VO_2_) resulted in faster phosphocreatine resynthesis during SIE_ARDS_. Further studies should verify, among others, the effect of ARDS_V_ breathing on the oxygenation and deoxygenation levels of working muscles by means of near-infrared spectroscopy and verify the impact on the rate of phosphocreatine resynthesis or the removal of metabolites such as inorganic phosphate during SIE.

The results of our own research indicate that breathing with air of altered composition increased blood P_ET_CO_2_ and pCO_2_, but no hypercapnia occurred. The higher mean P_ET_CO_2_ (ca. 15%) maintained during the main part of SIE_ARDS_ and the higher mean pCO_2_ after the fifth and sixth bout caused a decrease in the average blood pH and an increase in its HCO_3_^–^ concentration. In other experiments where ARDS_V_ was applied, higher P_ET_CO_2_ values were obtained than in own research [33,34] despite lower volumes (500–600-mL). This may be due to the different nature of the effort, the individual tolerance to the investigated individuals to CO_2_, vital lung capacity, and lower sensitivity of chemoreceptors in stimulating the ventilation response and CO_2_ elimination [35]. It is not surprising that blood pH was lower during ARDS_V_ breathing; it proves the occurrence of respiratory acidosis, as reported by Smołka et al. [15]. Breathing through a much smaller ARDS volume (350-mL) induced by the airflow restriction mask with special adaptation piece to gas analyzer, also triggered respiratory acidosis (lower blood pH) by inhaling CO_2_ [8]. Respiratory acidosis probably also affects the acid–base balance at the muscular level, which may result in metabolic changes. Higher HCO_3_^–^ concentration was observed by Woorons et al. [36] during hypoventilation exercises, in which partial CO_2_ pressure increases. This phenomenon can be a source of adaptation, leading to delayed acidosis and improved buffering capacity; it can also be beneficial for pH regulation and the development of the ability to produce energy via anaerobic metabolism [37]. This is especially important because the reduction of energy available from anaerobic glycolysis, muscular H^+^ accumulation, and the increase in extracellular potassium are major fatigue regulators during high-intensity exercises such as maximum sprinting effort [28]. Detailed analysis requires further investigation at the muscular level, which we did not perform in this study.

The increased blood partial CO_2_ pressure and H^+^ concentration induced higher mean VE during SIE_ARDS_. This was probably due to the stimulation of peripheral and central chemoreceptors by CO_2_ [38,39]. However, no respiratory alkalosis was observed. Higher VE occurred through higher VT, which is in line with other studies [11,16,33,34]. The higher mean VE in SIE_ARDS_ resulted in increased mean VO_2_. In a study by Jensen et al. [16], no VO_2_ increase was reported during effort with 500-mL ARDS_V_. In turn, other studies inform that the energy cost of respiratory muscle work was higher when breathing with ARDS_V_ [34], with a training mask [8], and with air enriched with CO_2_ [6], which resulted from greater muscle involvement. Similar interpretation was provided by Woorons et al. [40], who used hypoventilation during exercise. Another factor contributing to higher VO_2_ in SIE_ARDS_ may be related to the right-shift of the hemoglobin dissociation curve during acidosis, which is in accordance with the Bohr effect [41]. This increases the O_2_ diffusion gradient between capillaries and muscle cells, leading to a greater use of O_2_ in the cellular metabolism. The phenomenon confirms the statistically significantly lower P_ET_O_2_ during SIE_ARDS_. We are aware that measuring hemodynamic parameters of heart performance such as stroke volume and cardiac output would provide more evidence to explain the impact of ARDS_V_ on oxygen transport and VO_2_ during a single SIE session. Differences are likely to exist as mean HR was similar in both protocols. Therefore, other factors may also have been responsible for higher VO_2_ and should be assessed in further studies.

Among the main VE regulators during exercise, there is the so-called central neural drive (central command) [21]. Moreover, it has been suggested that the central command preferentially regulates Rf and not VT [21]. Rf is also a sensory signal for RPE [42] and provides its neurophysiological explanation. Breathing through the aforementioned training mask limiting the air flow with special valves and increasing ARDS by 350-mL resulted in lower Rf; however, the authors did not report RPE [8]. In our study, we did not identify any differences in Rf or RPE when ARDS_V_ breathing was applied. Thus, the SIE_ARDS_ session was not perceived as more difficult, which is of great importance for its implementation in regular training in a variety of populations. Similar findings were reported by Jung et al. [43], where RPE did not differ between normal breathing and respiration with ETM during continuous cycling (50 and 70% VO_2_max). Unfortunately, we did not measure other psychological characteristics, such as the sense of pleasure. Nonetheless, protocols perceived as more pleasant are more likely to be chosen and performed by practitioners [44].

It is interesting that after both SIE protocols, there was no respiratory muscle fatigue as observed after performing maximum effort, e.g., a progressive test [45]. This suggests that ARDS_V_ breathing is not a limiting factor for respiratory system effort despite the changed ventilation pattern—all the more so because the average ventilation did not exceed 60% of the maximum value. The applied snorkel of 1200-mL volume and 2.5-cm diameter does not seem to be a stimulus that could affect additional RMT. It is consistent with the last opinion by Shei [46], who explains recommendations to stimulus respiratory muscle training adaptations. A tube with a smaller diameter can increase resistance and engage the respiratory muscles to a greater extent. This corroborates the postulates by Illi et al. [47] indicating that increased respiratory muscle work induced by additional respiratory resistance improves endurance performance through RMT. This should become a subject of separate studies on long-term adaptation due to SIT with ARDS_V_.

Higher VO_2_ may have influenced changes in cellular metabolism. However, we did not notice any differences in La^−^ concentration between the SIE protocols, although there is a tendency for it to decrease in SIE_ARDS_. Several studies have demonstrated that increased blood partial pressure of CO_2_ can lower the release of La^−^ from muscles [23,24]. On the other hand, Smołka et al. [15] did not report differences in La^−^ concentration when the study participants breathed through 1200-mL ARDS_V_ during a 30-min exercise at 60% VO_2_max. We cannot entirely rule out the possibility that SIE_ADRSV_ led to a lower muscle La^−^ production than that during SIE_STD_. It is well known that blood La^−^ concentration does not mirror muscle La^−^ concentration. Solving this problem demands tissue analyses, which were not performed in this study.

It can be considered that the outcome of this study was limited by the fact that the participants were not blinded and that the differences in the amount of work performed and the lack of differences in RPE resulted from the placebo effect. Similar observations were earlier reported by Woorons et al. [37,40], who investigated the impact of hypoventilation. A corresponding problem refers to ARDS_V_, as it is impossible to conduct single- or double-blind trials. However, although a psychological effect cannot be ruled out in this study, it should be noted that the participants were not aware of or received no information on the potential impact of the tested method. It also seems very interesting to examine whether ARDS_V_ breathing modifies the contribution of particular energy systems in satisfying the metabolic needs of working muscles during maximum sprinting efforts.

## 5. Conclusions

The application of 1200-mL of ARDS_V_ during SIE is a simple method to induce acute stronger physiological responses and changes in the acid–base balance. SIE_ARDS_ sessions are not perceived as more difficult and can provide an alternative to the currently known training protocols. To stimulate RMT, other parameters of the applied device should be considered, e.g., increasing of the ARDS_V_ snorkel diameter.

## Figures and Tables

**Table 1:** Participants’ characteristics ($\bar{x}\pm \mathrm{SD}$).

Variables	Values
Age (years)	22.4 ± 3.9
Body height (cm)	181.0 ± 7.9
Body mass (kg)	77.1 ± 10.8
Physical activity (h per week)	7.5 ± 1.5
Systolic blood pressure (mm Hg)	124 ± 10
Diastolic blood pressure (mm Hg)	70 ± 8
FVC (l)	6.9 ±1.0
FEV_1_ (l)	5.1 ± 0.9
FEV_1_ · FVC^−1^ (%)	74.1 ± 9.9
PIF (l · s^−1^)	3.2 ± 1.5
PEF (l · s^−1^)	9.4 ± 1.8

FVC—forced vital capacity, FEV_1_—forced expiratory volume in 1 s, FEV_1_ · FVC^−^^1^—Tiffeneau index, PIF—peak inspiratory flow, PEF—peak expiratory flow.

**Table 2:** Comparison of mean ($\bar{x}$ ± SD) physiological responses between both SIE protocols.

Parameters	SIE_STD_	SIE_ARDS_	*p*-Value	*t*-Test
FiO_2_ (%)	20.8 ± 0.04	20.6 ± 0.13 *	<0.001	4.94
FiCO_2_ (%)	0.08 ± 0.03	0.19 ± 0.09 *	<0.01	4.36
P_ET_O_2_ (mm Hg)	112.7 ± 4.3	108.0 ± 3.6 *	<0.001	6.02
P_ET_CO_2_ (mm Hg)	32.6 ± 3.3	37.5 ± 2.7 *	<0.001	8.82
Rf (breath · min^−1^)	31.0 ± 3.4	32.6 ± 4.1	0.10	1.83
VT (L)	2.5 ± 0.4	2.7 ± 0.4 *	<0.001	4.96
VE (L · min^1^)	76.3 ± 12.1	86.4 ± 8.6 *	<0.01	3.50
VO_2_ (mL∙kg^−1^∙min^−1^)	27.8 ± 2.4	36.5 ± 5.0 *	<0.001	6.77
VE · VO_2_^−1^	36.9 ± 5.1	31.8 ± 3.5 *	<0.001	4.57
VE · VCO_2_^−1^	35.4 ± 4.7	30.5 ± 3.8 *	<0.01	3.88
Ti (s)	0.96 ± 0.12	0.93 ± 0.14	0.20	1.39
Te (s)	1.05 ± 0.12	0.97 ± 0.12	0.09	1.87
Ttot (s)	2.01 ± 0.22	1.90 ± 0.25	0.10	1.81
Ti · Ttot^−1^	0.48 ± 0.02	0.49 ± 0.02	0.11	1.74
HR (beats∙min^−1^)	153 ± 12.3	151 ± 8	0.57	0.59

FiO_2_—fraction of inspired oxygen, FiCO_2_—fraction of inspired carbon dioxide, P_ET_O_2_—end-tidal partial pressure of oxygen, P_ET_CO_2_—end-tidal partial pressure of carbon dioxide, Rf—respiratory frequency, VT—tidal volume, VE—respiratory minute ventilation, VO_2_—oxygen uptake, VE · VO_2_^−1^—ventilatory equivalent for oxygen, VE · VCO^−1^—ventilatory equivalent for carbon dioxide, Ti—time of inspiration, Te—time of expiration, Ttot—total time of the respiratory cycle, Ti · Ttot^−1^—the ratio of inspiration time and respiratory cycle time, HR—heart rate. * Statistically significant difference between the SIE protocols.
